# Biallelic *PTPMT1* variants disrupt cardiolipin metabolism and lead to a neurodevelopmental syndrome

**DOI:** 10.1093/brain/awae268

**Published:** 2024-08-30

**Authors:** Micol Falabella, Chiara Pizzamiglio, Luis Carlos Tabara, Benjamin Munro, Mohamed S Abdel-Hamid, Ece Sonmezler, William L Macken, Shanti Lu, Lisa Tilokani, Padraig J Flannery, Nina Patel, Simon A S Pope, Simon J R Heales, Dania B H Hammadi, Charlotte L Alston, Robert W Taylor, Hanns Lochmuller, Cathy E Woodward, Robyn Labrum, Jana Vandrovcova, Henry Houlden, Efstathia Chronopoulou, Germaine Pierre, Reza Maroofian, Michael G Hanna, Jan-Willem Taanman, Semra Hiz, Yavuz Oktay, Maha S Zaki, Rita Horvath, Julien Prudent, Robert D S Pitceathly

**Affiliations:** Department of Neuromuscular Diseases, University College London Queen Square Institute of Neurology, London WC1N 3BG, UK; Department of Neuromuscular Diseases, University College London Queen Square Institute of Neurology, London WC1N 3BG, UK; NHS Highly Specialised Service for Rare Mitochondrial Disorders, Queen Square Centre for Neuromuscular Diseases, The National Hospital for Neurology and Neurosurgery, London WC1N 3BG, UK; Medical Research Council Mitochondrial Biology Unit, University of Cambridge, Cambridge CB2 0XY, UK; Department of Clinical Neurosciences, University of Cambridge, Cambridge CB2 3EB, UK; Medical Molecular Genetics Department, Human Genetics and Genome Research Institute, National Research Centre, Cairo 12622, Egypt; Department of Medical Biology, Faculty of Medicine, Dokuz Eylül University, Izmir 35340, Turkey; Department of Neuromuscular Diseases, University College London Queen Square Institute of Neurology, London WC1N 3BG, UK; NHS Highly Specialised Service for Rare Mitochondrial Disorders, Queen Square Centre for Neuromuscular Diseases, The National Hospital for Neurology and Neurosurgery, London WC1N 3BG, UK; Department of Neuromuscular Diseases, University College London Queen Square Institute of Neurology, London WC1N 3BG, UK; Medical Research Council Mitochondrial Biology Unit, University of Cambridge, Cambridge CB2 0XY, UK; Neurogenetics Unit, Rare and Inherited Disease Laboratory, North Thames Genomic Laboratory Hub, London WC1N 3BH, UK; Genetics and Genomic Medicine, UCL Great Ormond Street Institute of Child Health, London WC1N 1EH, UK; Genetics and Genomic Medicine, UCL Great Ormond Street Institute of Child Health, London WC1N 1EH, UK; Neurometabolic Unit, The National Hospital for Neurology and Neurosurgery, London WC1N 3BG, UK; Genetics and Genomic Medicine, UCL Great Ormond Street Institute of Child Health, London WC1N 1EH, UK; Neurometabolic Unit, The National Hospital for Neurology and Neurosurgery, London WC1N 3BG, UK; Genetics and Genomic Medicine, UCL Great Ormond Street Institute of Child Health, London WC1N 1EH, UK; Neurometabolic Unit, The National Hospital for Neurology and Neurosurgery, London WC1N 3BG, UK; Wellcome Centre for Mitochondrial Research, Translational and Clinical Research Institute, Faculty of Medical Sciences, Newcastle University, Newcastle upon Tyne NE1 7RU, UK; Wellcome Centre for Mitochondrial Research, Translational and Clinical Research Institute, Faculty of Medical Sciences, Newcastle University, Newcastle upon Tyne NE1 7RU, UK; NHS Highly Specialised Service for Rare Mitochondrial Disorders of Adults and Children, Newcastle upon Tyne Hospitals NHS Foundation Trust, Newcastle upon Tyne NE1 4LP, UK; Wellcome Centre for Mitochondrial Research, Translational and Clinical Research Institute, Faculty of Medical Sciences, Newcastle University, Newcastle upon Tyne NE1 7RU, UK; NHS Highly Specialised Service for Rare Mitochondrial Disorders of Adults and Children, Newcastle upon Tyne Hospitals NHS Foundation Trust, Newcastle upon Tyne NE1 4LP, UK; Children's Hospital of Eastern Ontario Research Institute, University of Ottawa, Ottawa ON K1H 8L1, Canada; Division of Neurology, Department of Medicine, The Ottawa Hospital, Ottawa ON K1Y 4E9, Canada; Department of Neuropediatrics and Muscle Disorders, Medical Center—University of Freiburg, Faculty of Medicine, Freiburg 79106, Germany; Centro Nacional de Análisis Genómico (CNAG), Barcelona Institute of Science and Technology (BIST), Barcelona 08003, Spain; Neurogenetics Unit, Rare and Inherited Disease Laboratory, North Thames Genomic Laboratory Hub, London WC1N 3BH, UK; Neurogenetics Unit, Rare and Inherited Disease Laboratory, North Thames Genomic Laboratory Hub, London WC1N 3BH, UK; Department of Neuromuscular Diseases, University College London Queen Square Institute of Neurology, London WC1N 3BG, UK; Department of Neuromuscular Diseases, University College London Queen Square Institute of Neurology, London WC1N 3BG, UK; Department of Inherited Metabolic Disease, Division of Women's and Children's Services, University Hospitals Bristol NHS Foundation Trust, Bristol BS1 3NU, UK; Department of Inherited Metabolic Disease, Division of Women's and Children's Services, University Hospitals Bristol NHS Foundation Trust, Bristol BS1 3NU, UK; Department of Neuromuscular Diseases, University College London Queen Square Institute of Neurology, London WC1N 3BG, UK; Department of Neuromuscular Diseases, University College London Queen Square Institute of Neurology, London WC1N 3BG, UK; NHS Highly Specialised Service for Rare Mitochondrial Disorders, Queen Square Centre for Neuromuscular Diseases, The National Hospital for Neurology and Neurosurgery, London WC1N 3BG, UK; Department of Clinical and Movement Neurosciences, University College London Queen Square Institute of Neurology, London WC1N 3BG, UK; Izmir Biomedicine and Genome Center, Dokuz Eylul University Health Campus, Izmir 35340, Turkey; Department of Pediatric Neurology, Faculty of Medicine, Dokuz Eylül University, Izmir 35340, Turkey; Department of Medical Biology, Faculty of Medicine, Dokuz Eylül University, Izmir 35340, Turkey; Izmir Biomedicine and Genome Center, Dokuz Eylul University Health Campus, Izmir 35340, Turkey; Clinical Genetics Department, Human Genetics and Genome Research Institute, National Research Centre, Cairo 12311, Egypt; Department of Clinical Neurosciences, University of Cambridge, Cambridge CB2 3EB, UK; Medical Research Council Mitochondrial Biology Unit, University of Cambridge, Cambridge CB2 0XY, UK; Department of Neuromuscular Diseases, University College London Queen Square Institute of Neurology, London WC1N 3BG, UK; NHS Highly Specialised Service for Rare Mitochondrial Disorders, Queen Square Centre for Neuromuscular Diseases, The National Hospital for Neurology and Neurosurgery, London WC1N 3BG, UK

**Keywords:** cardiolipin, mitochondria, mitochondrial dynamics, primary mitochondrial disease, neurodevelopmental syndrome

## Abstract

Primary mitochondrial diseases (PMDs) are among the most common inherited neurological disorders. They are caused by pathogenic variants in mitochondrial or nuclear DNA that disrupt mitochondrial structure and/or function, leading to impaired oxidative phosphorylation (OXPHOS). One emerging subcategory of PMDs involves defective phospholipid metabolism. Cardiolipin, the signature phospholipid of mitochondria, resides primarily in the inner mitochondrial membrane, where it is biosynthesized and remodelled via multiple enzymes and is fundamental to several aspects of mitochondrial biology. Genes that contribute to cardiolipin biosynthesis have recently been linked with PMD. However, the pathophysiological mechanisms that underpin human cardiolipin-related PMDs are not fully characterized.

Here, we report six individuals, from three independent families, harbouring biallelic variants in *PTPMT1*, a mitochondrial tyrosine phosphatase required for *de novo* cardiolipin biosynthesis. All patients presented with a complex, neonatal/infantile onset neurological and neurodevelopmental syndrome comprising developmental delay, microcephaly, facial dysmorphism, epilepsy, spasticity, cerebellar ataxia and nystagmus, sensorineural hearing loss, optic atrophy and bulbar dysfunction. Brain MRI revealed a variable combination of corpus callosum thinning, cerebellar atrophy and white matter changes.

Using patient-derived fibroblasts and skeletal muscle tissue, combined with cellular rescue experiments, we characterized the molecular defects associated with mutant *PTPMT1* and confirmed the downstream pathogenic effects that loss of PTPMT1 has on mitochondrial structure and function. To further characterize the functional role of PTPMT1 in cardiolipin homeostasis, we created a ptpmt1 knockout zebrafish. This model had abnormalities in body size, developmental alterations, decreased total cardiolipin levels and OXPHOS deficiency.

Together, these data indicate that loss of *PTPMT1* function is associated with a new autosomal recessive PMD caused by impaired cardiolipin metabolism, highlighting the contribution of aberrant cardiolipin metabolism towards human disease and emphasizing the importance of normal cardiolipin homeostasis during neurodevelopment.

## Teaser

PTPMT1 is crucial for biosynthesis of the signature mitochondrial phospholipid, cardiolipin. Falabella *et al.* show that loss of *PTPMT1* function is associated with an autosomal recessive primary mitochondrial disease, highlighting the importance of cardiolipin homeostasis in neurodevelopment.

## Introduction

Primary mitochondrial diseases (PMDs) are a heterogeneous group of genetic disorders with a prevalence of approximately 1 in 4300.^1^ They may have severe neuromuscular and multisystem manifestations and can reduce lifespan in children and adults.^2^ PMDs are caused by pathogenic variants in either mitochondrial DNA (mtDNA) or nuclear DNA (nDNA) that lead to defects in oxidative phosphorylation (OXPHOS). An emerging class of PMDs involves impaired phospholipid metabolism.^3,4^ Cardiolipin is a mitochondria-specific glycerophospholipid that comprises 10%–15% of the total mitochondrial phospholipid content. It is primarily located within the inner mitochondrial membrane (IMM), where it is central to several aspects of mitochondrial biology, including mitochondrial membrane architecture, OXPHOS and the stabilization of supercomplexes.^5^ In addition, cardiolipin contributes to mitochondrial fission and fusion dynamics, thus preserving the mitochondrial network^6^ and ensuring an appropriate response to cellular metabolic demands.^7,8^

Molecular defects in genes involved in cardiolipin biosynthesis and remodelling have been associated with several human diseases. Pathogenic variants in the acyltransferase tafazzin (*TAZ*) gene were the first to be reported and are associated with Barth syndrome,^9,10^ an ultra-rare disorder characterized by cardiomyopathy, skeletal myopathy, growth delay and neutropenia. Other monogenic cardiolipin disorders include Sengers syndrome (*AGK*),^11^ cerebellar ataxia and peripheral neuropathy (*PNPLA8*),^12,13^ dilated cardiomyopathy with ataxia syndrome (*DNAJC19*)^14^ and MEGDEL syndrome (*SERAC1*).^15^ In 2022, two cardiolipin biosynthesis genes, cardiolipin synthase 1 (*CRLS1*) and mitochondrial translocator assembly and maintenance homolog 41 (*TAMM41*), were linked to human diseases^16,17^ with neurological, cardiac and skeletal muscle manifestations.

Protein tyrosine phosphatase mitochondrial 1 (PTPMT1) is a highly conserved mitochondrial tyrosine phosphatase that plays a crucial role in *de novo* cardiolipin biosynthesis.^18-20^ It localizes to the matrix leaflet of the IMM via an N-terminal signal sequence,^21^ where it dephosphorylates phosphatidylglycerol phosphate to phosphatidylglycerol, an essential intermediate within the cardiolipin biosynthetic pathway. Total body ablation of *Ptpmt1* is embryonically lethal, suggesting the protein is fundamental for cardiolipin biosynthesis and cellular survival during development.^19,22^ Notably, *in vivo* studies using a conditional knockout in the heart, skeletal muscle and brain have shown that PTPMT1 is essential for cardiac and skeletal muscle physiology^23^; reduced levels are associated with impaired cellular proliferation and aberrant postnatal development,^24-26^ emphasizing the crucial role of PTPMT1 in mitochondrial aerobic metabolism and embryonic development. Although PTPMT1 has been associated with defects in mitochondrial activity in several *in vitro* and *in vivo* models, its pathogenic role in humans has not yet been described.

Here, we report six individuals from three unrelated families presenting with a neonatal/infantile onset neurological and neurodevelopmental syndrome associated with cardiolipin abnormalities resulting from novel biallelic variants in the *PTPMT1* gene (GenBank: NM_175732.3). Using patient-derived fibroblasts and skeletal muscle tissue, combined with cellular rescue experiments, we have characterized the molecular defects caused by mutant *PTPMT1* and validated the pathogenicity of the reported variants. These studies revealed that loss of PTPMT1 impairs cardiolipin biosynthesis, OXPHOS and mitochondrial morphology, thus establishing *PTPMT1* as a new cardiolipin-related disease gene and implicating cardiolipin in neurodevelopment.

## Materials and methods

### Next-generation sequencing analysis

Next-generation sequencing was undertaken via the whole genome or exome, depending on local availability (Supplementary Table 1). Families underwent whole exome or whole genome sequencing via the 100 000 Genomes Project (Family 1) and the Consequitur Project (Family 2)^27,28^ or clinical diagnostic testing (Family 3). During the initial analysis, data were filtered for variants in known childhood onset disease genes (https://panelapp.genomicsengland.co.uk/panels/486/) following a recessive, *de novo* or X-linked inheritance pattern and classified according to American College of Medical Genetics (ACMG) criteria.^29^ However, no likely pathogenic or pathogenic variants were identified. Data were also annotated with ClinVar disease status, and no relevant likely pathogenic or pathogenic variants were identified. Additionally, copy number variant analysis was undertaken using various methodologies depending on local processes (Supplementary Table 2) and did not identify any causative variants. Finally, a research-based agnostic approach was taken, focusing on coding variants and variants with a putative effect on splicing. Data were filtered for rare heterozygous *de novo*, rare biallelic and rare X chromosome variants in the affected individuals. Minor allele frequency cut-offs were assigned based on gnomAD v3 data (0 for *de novo* variants and <0.01 for biallelic variants, <0.01 X-linked variants and 0 for hemizygous X chromosome variants). Variants were annotated using Ensembl Variant Effect Predictor (VEP) with CADD (Combined Annotation Dependent Depletion), SIFT, PolyPhen2 and SpliceAI scores. We prioritized coding and putative splice variants with a CADD score of >20 (scale range: 0 = potentially benign; 48 = potentially pathogenic) or SpliceAI delta scores of >0.2 (scale range from 0 to 1). Details of variant filtering and prioritization are summarized in Supplementary Table 3.

### RNA sequencing

RNA was isolated from cultured fibroblasts obtained from a skin biopsy of Subjects S1 and S2. Sequencing was outsourced to UCL Genomics, London and was performed on an Illumina NovaSeq with 100 bp paired-end reads following library preparation using Kapa mRNA Hyper Prep. Samples were sequenced to a depth of 100 million reads. FASTQ files were aligned using Spliced Transcripts Alignment to a Reference (STAR) software.^30^ Quality control was performed via MultiQC on STAR and FastQC data.^31^ BAM files and Sashimi plots were visualized using Integrative Genomics Viewer (IGV). Differential analysis of pseudoaligned data was performed using the Sleuth package following processing using Kallisto.^32^

### Generation of zebrafish CRISPR/Cas9 *ptpmt1* knockout

To generate a stable zebrafish mutant line, a standard Clustered Regularly Interspaced Short Palindromic Repeats (CRISPR)/Cas9 mutagenesis protocol was used.^33^ One single guide RNA (sgRNA) was designed with the CHOPCHOP webtool^34^ using the zebrafish genome assembly, danRer11/GRCz11, targeting exon 3 of zebrafish *ptpmt1* (GCAAGACTGGGAACACCTGT). Once the sgRNA template was synthesized and sgRNA transcribed, the injection mix was prepared as follows: sgRNA (300 ng/µl), 2 μM Cas9 protein (with nuclear localization sequence) (New England Biolabs), Cas9 buffer (New England Biolabs), 2 M KCl and 0.05% phenol red and heated at 37°C for 5 min. Using a microinjector, 1 nl of injection mix was injected into the yolk/cell boundary of newly fertilized (<20 min post-fertilization) zebrafish embryos (TLF strain). Once injected, embryos were raised following standard procedures for zebrafish husbandry as the founder generation (F0). Once the F0 reached sexual maturity, they were outcrossed with wild-type adults, and a sample of resulting embryos was genotyped via Sanger sequencing to confirm germline transmission of mutations in *ptpmt1*. Once confirmed, F1 fish were then raised to maturity and genotyped via Sanger sequencing to identify mutations of interest. F1 fish with the same mutation were identified and in-crossed to produce the F2 generation and the stable line.

### Zebrafish genotyping

gDNA was extracted using a HotSHOT extraction method. Tissue was lysed in 100 µl (fin cip) or 50 µl [larvae <5 days post-fertilization (dpf)] of 50 mM NaOH and heated at 95°C for 20 min, followed by neutralization with 1/10th volume of 1 M Tris-HCl. DNA concentration was quantified using a NanoDrop 2000 spectrophotometer (Thermo Fisher Scientific). The region surrounding the mutation site was amplified via PCR using the following primers: *ptpmt1* Forward, GGCCTTATTGTAAAGTGTTCCCT, *ptpmt1* Reverse, TATAGACACTGCTGCCCTGTTC. PCR products were subsequently analysed via Sanger sequencing (in-house service) or digested with the restriction endonuclease *Sex*AI (New England Biolabs) at 37°C, which specifically targets and digests the mutant sequence. Digested products were analysed via agarose gel electrophoresis.

### Zebrafish morphological assessment

Zebrafish were imaged at 19 dpf using a stereo microscope with a Dino-lite AM7025X eyepiece camera. Morphological parameters were quantified using FIJI software.^35^ Standard length was taken as the distance from the mouth to the tip of the tail, not including caudal fin. Head area was taken as the total area anterior of the height at the nape.

### Blue-native polyacrylamide gel electrophoresis and in-gel activity assays

Zebrafish were lysed at 19 dpf in 0.5% *n*-dodecyl-β-D-maltoside, 1 M 6-aminocaproic acid, 50 mM Bis-Tris (pH 7.0), 1 mM phenylmethane-sulfonyl fluoride, 1 μg/ml leupeptin and 1 μg/ml pepstatin A on ice for 15 min and centrifuged at 13 000*g* for 15 min at 4°C. Protein lysates were quantified using the BCA Protein Assay Kit (Thermo Fisher Scientific, Cat. No. 23225) and the supernatant diluted into an equal protein concentration and 1/6 volume of 1 M 6-aminocaproic acid, 5% Serva blue G (Serva Electrophoresis, Cat. No. 3505003). Native samples were separated using 3%–12% native polyacrylamide gels with a 3% stacking gel (23.8 μg of protein/lane) as described by Schägger.^36^ Blue-native polyacrylamide gels were transferred to Immobilon-PSQ polyvinylidene fluoride (PVDF) membranes (Millipore, Cat. No. 1SEQ00010).^37^ The membranes were rinsed three times with methanol to remove residual dye, then blocked with 10% skimmed milk powder in PBS and incubated overnight at 4°C with primary antibodies against NDUFS3 (Abcam, Cat. No. ab14711, 1:300 dilution); MTCO1 (Abcam, Cat. No. ab14705, 1:1000 dilution) or SDHA (Proteintech, Cat. No. 14865-1-AP, 1:3000 dilution) diluted in 3% bovine serum albumin in PBS-Tween 20. Membranes were incubated with anti-rabbit (Dako, Cat. No. P0448, 1:4000) or anti-mouse (Promega, Cat. No. W402B, 1:3000 dilution) horse radish peroxidase-conjugated secondary antibodies for 1 h. Images were detected with the Bio-Rad Chemidoc MP Imaging System and quantified using Bio-Rad Image Lab 5.1 software. Uncropped images are shown in Supplementary Fig. 7.

For the Complex I (NADH dehydrogenase) in-gel activity assay, the blue-native gel (35.7 μg of protein/lane) was stained with 30 ml 2 mM Tris·HCl (pH 7.4), 3 mg NADH and 75 mg of nitroblue tetrazolium (NBT) for 1 h at 37°C with gentle shaking. The reaction was stopped by incubating the gel with 10% acetic acid and 40% methanol overnight, as previously described.^38^ Finally, the gel was rinsed with ddH_2_O and imaged. For the Complex V in-gel activity assay, samples (11.4 μg of protein/lane) were mixed with 1/10 of a volume of 50% glycerol and 1/10 of a volume of 0.01% Ponceau S and separated using 3%–12% clear native polyacrylamide gels with a 3% stacking gel (10 μg of protein/lane) and a cathode buffer composed of 50 mM tricine, 15 mM Bis-Tris (pH 7.0), 0.05% Triton X-100 and 0.05% sodium deoxycholate.^39^ The gel was incubated with 50 ml 34 mM Tris, 270 mM glycine, 14 mM MgSO_4_, 0.2% Pb(NO_3_)_2_ and 8 mM ATP (pH 7.8) for 1 h at 37°C with gentle shaking, rinsed with ddH_2_O and briefly incubated with 1% ammonium sulfide [S(NH_4_)_2_] solution.^40^ The reaction was stopped with 50% methanol, the gel rinsed with ddH_2_O and imaged. Images were quantified using ImageJ v.2.0.0 software (National Institutes of Health). To determine the linear dynamic range for the in-gel activity assays, two dilution series were performed using protein extracted from control (CTR) zebrafish at 19 dpf (Supplementary Fig. 6C and D). Uncropped images are shown in Supplementary Fig.8.

### Statistical analyses

Statistical analysis was performed by two-way ANOVA test, Mann–Whitney U-test (two-tailed), or unpaired *t*-test (two-tailed) using GraphPad Prism 8 software (GraphPad Software Inc., CA). All data are expressed as mean ± standard error of the mean (SEM) or standard deviation (SD). For qPCR experiments, the value of each biological replicate represents the mean of three technical triplicate reactions. Significance values are expressed as follows: *P < 0.05, **P < 0.01, ***P < 0.001 and ****P < 0.0001. Figures were created using BioRender.com.

## Results

### Identification of candidate pathogenic *PTPMT1* variants

**Figure 1:**
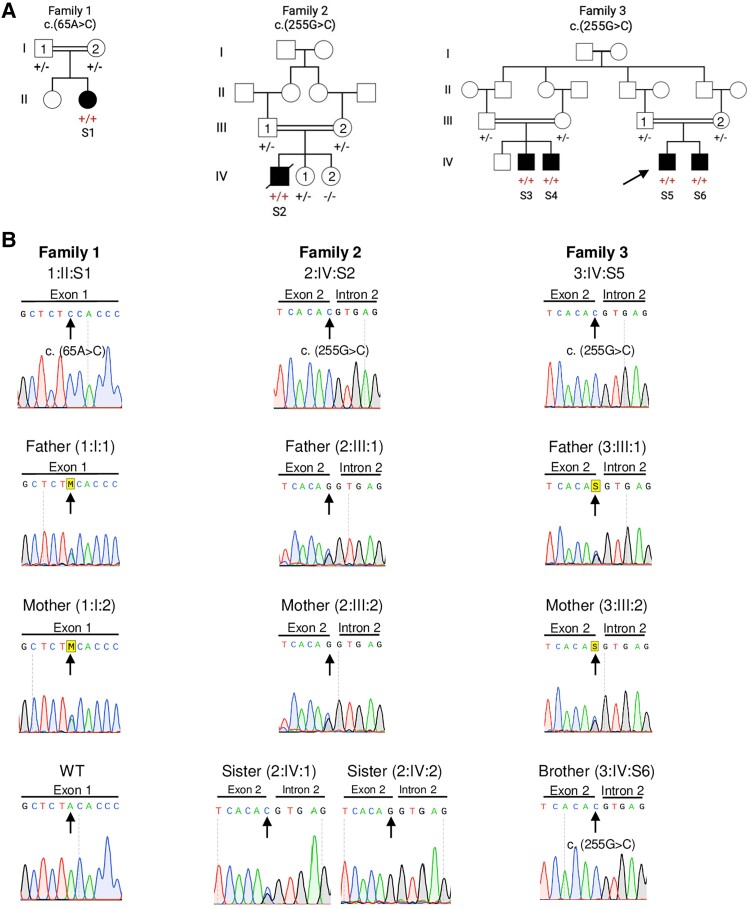
**Family pedigrees and details of *PTPMT1* variants.** (**A**) Pedigrees of the three unrelated families showing consanguinity (double horizontal lines). Affected subjects (Subjects S1–S6) are represented by filled circles for females and filled squares for males. Arrow indicates proband (Subject S5) of Family 3. (**B**) Chromatograms from Sanger sequencing of *PTPMT1* (NM_175732.3) genotypes for probands, parents and siblings of Families 1–3.

**Figure 2:**
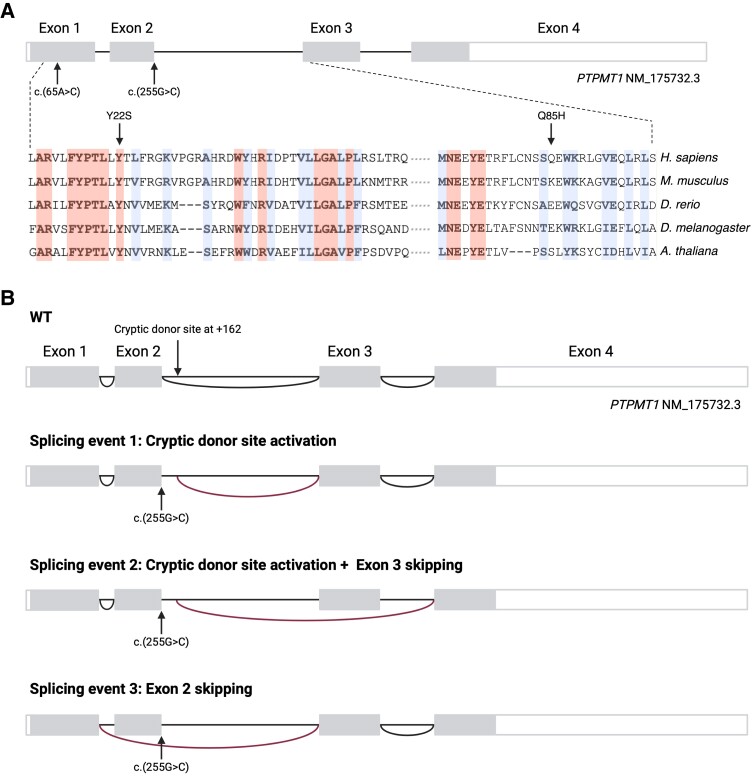
***PTPMT1* variants and gene structure.** (**A**) Schematic representation of the *PTPMT1* gene showing the localization of variants c.65A>C and c.255G>C and their effects on protein sequence. PTPMT1 protein sequences alignment, indicating identical and highly conserved residues. (**B**) RNA-sequencing analysis of the *PTPMT1* c.255G>C splicing variant. Schematics of normal PTPMT1 splicing (*top*) and the three aberrant splicing events associated with the c.255G>C variant.

We identified three families, including six affected individuals with biallelic variants in *PTPMT1*. Pedigrees are displayed in Fig. 1A. Given no pathogenic variants in known disease-causing genes were identified, a research re-evaluation was performed to identify rare *de novo* and biallelic variants in the affected individuals. Through this filtering strategy, we identified candidate variants in *PTPMT1* (other shortlisted candidate variants are summarized and interpreted in Supplementary Table 4): a homozygous missense variant in Family 1 and a homozygous variant, predicted to have both missense and splicing effects (falling in the last nucleotide of exon 2, NM_175732.3; Table 1 and Fig. 2) in Families 2 and 3. Both variants were absent from gnomAD v3. All *PTPMT1* variants identified resided within a region of homozygosity and were confirmed by Sanger sequencing (Fig. 1B). Given the presence of the same variant in Families 2 and 3, we undertook a DNA microarray analysis to establish genetic relatedness. Analysis of DNA from Subjects S2 and S5 revealed that the two individuals shared a haplotype, suggesting some common ancestry between the two individuals, likely more distant than a second cousin relationship (Supplementary Fig. 1A).

**Table:** Table 1 Clinical, radiological, biochemical and molecular characteristics of six subjects with biallelic PTPMT1 variants

	Family 1	Family 2	Family 3			
Subject ID	S1	S2	S3 IV-2	S4 IV-3	S5 IV-4	S6 IV-5
**Genotype**	c.65A>C; p.Tyr22Ser	c.255G>C	c.255G>C			
**Demographics**						
Sex; age, years	F; 14	M; 16 (deceased)	M; 8	M; 4	M; 4	M; 1
Ethnicity	Kurdish	Turkish	Iraqi	Iraqi	Iraqi	Iraqi
**Clinical presentation**						
Age of onset	Birth	Birth	1 y 2 mo	1 y	1 y 3 mo	9 mo
Onset symptom	HIE Grade 2 with PPHN	Hypotonia, lack of eye contact	DD, nystagmus	DD, nystagmus	DD, nystagmus	DD, nystagmus
Disease progression^a^	Slow	Slow	Moderate	Moderate	Moderate	U (new onset)
Neuromuscular	Exercise intolerance	Hypotonia, bulbar dysfunction	Bulbar dysfunction	Bulbar dysfunction	Bulbar dysfunction	Bulbar dysfunction
Gastrointestinal	Chronic vomiting	–	Feeding difficulties	Feeding difficulties	Feeding difficulties	–
Hepatology	Elevated transaminases, hepatomegaly	–	–	–	–	–
Ophthalmology	–	VEPs: delayed P100-like latency on right	Optic nerve atrophy	Optic nerve atrophy	Optic nerve atrophy	–
Neurology	Seizures (onset 6 y), severe SNHL	Seizures (onset 6 mo), spasticity, cerebellar ataxia, SNHL	Nystagmus, cerebellar ataxia, spastic quadriparesis, head nodding	Nystagmus, cerebellar ataxia, hypertonia, head nodding	Nystagmus, cerebellar ataxia, hypertonia, head nodding	Nystagmus, cerebellar ataxia, head nodding
**Development**						
Global DD	Mild	Severe	Mild	Mild	Mild	Mild
Gross motor	Crawling (12 mo), walking (18 mo)	Crawling (5 y), never walked	Head support (4 mo), sitting (9 mo), standing supported (1 y 3 mo)	Head support (4 mo), sitting (10 mo), standing supported (1 y 2 mo)	Head support (4 mon), sitting (1 y), standing supported (1 ye 3 mon)	Sitting (9 mo)
Speech	15 words (2 years)	3–4 words (4 years)	Delayed	Delayed	Delayed	Delayed
Developmental regression	–	–	Y	Y	Y	–
Microcephaly	–	Y	Y	Y	Y	Y
Facial/body dysmorphisms	–	Hypertelorism, eye exotropia, large ears, high palatal arch, pectus carinatum	Bitemporal hollowing, upward palpebral fissures, epicanthic folds, prominent nose, long philtrum, low set large ears	Bitemporal hollowing, prominent nose, low set large ears	Sloping forehead, upward palpebral fissures, epicanthal folds, prominent nose, low set large ears	Upward palpebral fissures, upturned prominent nose, receded chin, low set large ears
**Blood tests**						
Venous lactate (mmol/l)^b^	1.2–4	1.14–1.69	2.44	2.78	3.11	1.78
CK	N	N	N	N	N	N
**Investigations**						
Brain MRI	Thin CC, small pons/medulla	Thin CC, progressive cerebellar atrophy, diffuse hypomyelination	Mild HS deep WM, dilatated lateral ventricles	Mild cortical atrophy, cerebellar atrophy, deep WM HS	Cerebellar atrophy, dilatated lateral ventricles, periventricular WM HS	N
Muscle biopsy^c^	CI deficiency^d^	N/A	N/A	N/A	N/A	N/A

– = symptom not present; CC = corpus callosum; CI = Complex I; CK = creatine kinase; DD = developmental delay; F = female; HC = head circumference; HIE = hypoxic-ischaemic encephalopathy; HS = high signal; M = male; N = normal; N/A = not available; PPHN = persistent pulmonary hypertension of the new-born; SD = standard deviation; SNHL = sensorineural hearing loss; U = unknown; VEP = visual evoked potential; WM = white matter; Y = yes.

^a^According to primary care provider.

^b^Normal values 0.5–2.2 mmol/l.

^c^Histology not available due to lack of tissue.

^d^CI: 0.053 nmol NADH oxidized/min/unit citrate synthase (reference range: 0.104 ± 0.036).

### Clinical presentations of subjects harbouring *PTPMT1* variants

**Figure 3:**
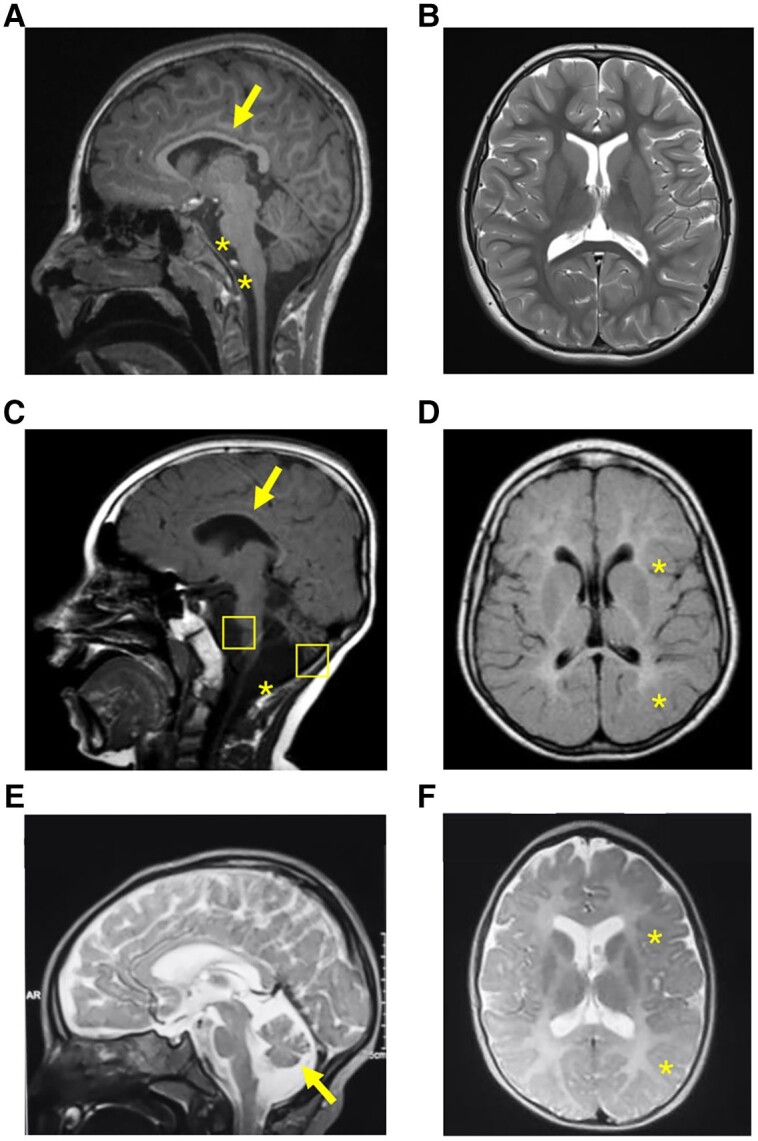
**MRI sections of the brain of the three probands harbouring biallelic *PTMT1* variants.** (**A**) T1 sequence, sagittal view. Evidence of thin corpus callosum (arrow), atrophy of pons and medulla (star). (**B**) T2 sequence, axial view. No white or grey matter changes were observed. (**C**) T1 sequence, sagittal view, showing hypoplasia of corpus callosum (arrow), cerebellar and brainstem atrophy (squares), and mega cisterna magna anomaly (asterisk). (**D**) T1 sequence, axial view, showing supratentorial white matter signal hyperintensity in keeping with hypomyelination. (**E**) T2 sequence, sagittal view, showing diffuse cortical atrophy, and cerebellar atrophy (arrow). (**F**) T2 sequence, axial view, showing areas of hypomyelination.

Subject S1 (Family 1; Fig. 1A) was the first child born to consanguineous Iraqi Kurdish parents at 39 weeks, following an emergency caesarean section for fetal distress. At birth, she showed signs of hypoxic-ischaemic encephalopathy (HIE) and persistent pulmonary hypertension (PPHN). Birth anthropometry was normal. As a neonate, she was diagnosed with sensorineural hearing loss and found to have hepatomegaly with elevated transaminases. She had persistent vomiting, requiring gastrostomy, which was removed at 8 years. Development was globally delayed; she crawled at 12 months, walked at 18 months, and at 24 months, had 15 words. She was later diagnosed with a mild learning disability and oppositional defiant behaviour disorder. Despite significant fatigue, muscle strength testing was normal. She developed nocturnal generalized tonic-clonic and absence seizures at 6 years. Anticonvulsants were initiated but discontinued aged 12 years after no further seizures were reported. Brain MRIs at 24 months and 8 years showed thinning of the corpus callosum and atrophy of the pons and medulla (Fig. 3A and B). There was normal appearance of the cerebellum and cerebral hemispheres, with no white or grey matter signal change. Lactate peak on brain MR spectroscopy at 8 years was normal. Respiratory chain enzyme analysis of muscle tissue showed a reduction (∼50%) in Complex I activity (0.053, reference range 0.104 ± 0.036). A complete report of muscle mitochondrial respiratory enzyme activities is provided in Supplementary Table 5.

Subject S2 (Family 2; Fig. 1A) was born at term to consanguineous Turkish parents. At birth, he was noted to be hypotonic. His birth weight was 2.6 kg. His development was globally delayed; he achieved head control at 2 years, started crawling/rolling at 5 years and never walked. Language was severely delayed; he had three to four words only at 4 years. He was noted to have microcephaly (44.2 cm, <−2 SD at 11.5 years) and dysmorphism, with hypertelorism, exotropia, large ears, high palatal arch and pectus carinatum. A severe learning disability was later diagnosed, and he was dependent for all daily activities. In addition, he had severe spasticity, cerebellar involvement, sensorineural hearing loss and mild bulbar dysfunction. He developed generalized seizures at 6 months, which terminated with valproic acid at 6 years. Serial brain MRIs at 12 months, 4 years and 8 years showed thinning of the corpus callosum, progressive cerebellar atrophy and diffuse hypomyelination (Fig. 3C and D). Brainstem auditory evoked potentials elicited no response bilaterally and there was delayed right P1 latency in visual evoked potentials. He died at the age of 16 years following a severe lower respiratory tract infection.

Family 3 was of Iraqi descent, with four affected males (Subjects S3–S6) derived from two branches, who were the fourth generation of the same grandparents (Fig. 1A). In both branches, parents were first cousins.

Subjects S3 and S4 were born at term with normal birth weights following uneventful pregnancies. Subject S3 exhibited global developmental delay with regression at 18 months. At 14 months, clinical examination revealed nystagmus, tetraparesis, spasticity and microcephaly [45.7 cm (−4.8 SD) at 18 months]. Brain MRI at 18 months showed mildly dilated lateral ventricles and white matter signal abnormalities, with a normal cerebellum. Subject S4 had mild developmental delay and microcephaly [45.0 cm (−3.9 SD) at 18 months]. At 12 months, there was nystagmus, spasticity and abnormal limb movement. Brain MRI at 4 years showed mild cortical atrophy, cerebellar atrophy and white matter signal changes.

The antenatal and perinatal history of Subject S5 was unremarkable [weight 3 kg (−0.9 SD), length 48 cm (−0.8 SD) and head circumference 34 cm (−0.8 SD)]. He presented with nystagmus, unsteadiness and head nodding at the age of 6 months. Motor skills were delayed, with head control at 4 months, sitting at 12 months and standing (supported) at 15 months. He could speak two words at 15 months. Developmental regression followed from 22 months, with an inability to stand, speak or react to simple commands. At 20 months, he was noted to have developed microcephaly [44.8 cm (−4.8 SD)] and was dysmorphic, with a sloping forehead, upward palpebral fissures, epicanthal folds, prominent nose and low-set large ears. He subsequently developed lower limb spasticity and abnormal limb movements (Supplementary Video 1). Pale optic discs were documented. There were no seizures or multisystemic manifestations. Metabolic screening of blood and urine was normal, apart from a mildly elevated plasma lactate of 3.11 mmol/l (reference range 0.5–2.2 mmol/l). Brain MRI at 14 months and 4 years showed progressive cerebellar atrophy, mild dilatation of the lateral ventricles and white matter signal changes (Fig. 3E and F).

Subject S6 was delivered by caesarean section with normal anthropometry at birth [weight 3.3 kg (−0.4 SD), length 47 cm (−1 SD) and head circumference 34 cm (−0.8 SD)]. There was mild developmental delay, with sitting at 9 months. Nystagmus and head nodding were reported at 12 months. By 12 months, he was noted to be microcephalic [43.0 cm (−2.8 SD)] and dysmorphic. Brain MRI was normal at 9 months.

### Molecular genetics and predicted effects on protein stability of *PTPMT1* variants

The *PTPMT1* (NM_175732.3):c.65A>C; p.Tyr22Ser missense variant identified in Subject S1 (Family 1) had a CADD score of 28 and was predicted to be deleterious and probably damaging by SIFT and PolyPhen-2, respectively. In addition, the Tyr22 residue is highly conserved across species, from human to *Arabidopsis thaliana* (Fig. 2A and Supplementary Fig. 2A). To understand the localization and effect of the missense variant c.65A>C on the PTPMT1 protein, AlphaFold was used to predict the structure of human PTPMT1 and identify the location of the affected amino acid residue.^41^ The Tyr22 residue, localized in the N-terminus domain of the protein, includes a highly conserved α-helix and is predicted to be involved in hydrogen bonding with Arg26 (Supplementary Fig. 2B and C). PTPMT1 localizes to the mitochondria via the N-terminal amino acids 1 to 37.^21^ We speculate that the p.Tyr22Ser substitution affects protein stability and correct localization of PTPMT1 within the IMM.

The missense/splice variant c.255G>C; p.Gln85His identified in Subjects S2 and S3–S5 had a CADD score of 35 and was predicted to have a deleterious effect on the *PTPMT1* mRNA (Supplementary Table 6) by other *in silico* prediction programs, including Splice AI Delta Score^42^ and MaxEntScan.^43^ We speculated that this variant is more likely to exert its effect through aberrant splicing rather than amino acid substitution as (i) the Gln to His substitution is predicted to be benign by the metapredictor REVEL (0.2)^44^; (ii) the region contains a high level of missense variation on gnomAD and hence may be missense tolerant; and (iii) the variant location (neighbouring an acceptor site) is likely to cause abnormal splicing and a loss-of-function effect, which is typically more deleterious than a substitution.

### *PTPMT1* c.255G>C is a complex splice variant

To assess the effects on transcripts of the homozygous *PTPMT1* c.255G>C variant detected in Families 2 and 3 (unrelated), we undertook RNA sequencing (RNA-seq) analysis in cultured fibroblasts of Subject S2. Notably, RNA-seq data showed that three aberrant splicing events occur as a consequence of the c.255G>C variant, relative to the MANE transcript NM_175732.3 (Fig. 2B and Supplementary Fig. 3A and B). First, a cryptic donor site in intron 2 is activated, resulting in the inclusion of 162 nucleotides. This introduces a stop codon at amino acid 105 (i.e. p.Ile105Ter). In the second event, the use of this cryptic donor site in intron 2 is accompanied by the skipping of exon 3 without the generation of a novel stop codon. Finally, the third splicing event results in exon 2 skipping without the use of the cryptic donor site. No effect on mRNA splicing was observed in the cultured fibroblasts of Subject S1 by RNA-seq analysis. Aberrant splicing led to reduced expression levels of *PTPMT1* mRNA in the cultured fibroblasts of Subject S2, compared to controls, because of aberrant splicing (Supplementary Fig. 3C). RNA-seq data were validated using qPCR analysis of *PTPMT1* mRNA relative expression in the cultured fibroblasts of Subjects S2 and S5 (Fig. 5A). There was extensive loss of *PTPMT1* transcripts in both cases (Fig. 5A), confirming that the c.255G>C variant is associated with multiple splicing events and activation of nonsense-mediated decay (NMD).

### Variants in *PTPMT1* affect total cardiolipin abundance

**Figure 4:**
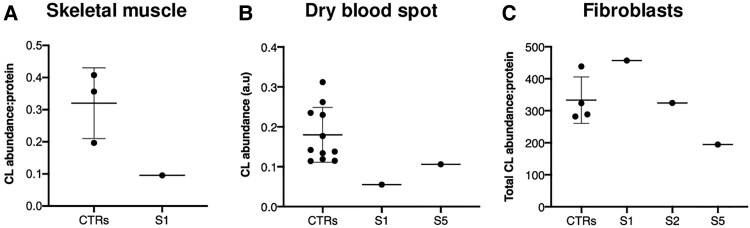
**Cardiolipin content and acyl chain composition in patient-derived tissues.** (**A**) The 72:8 cardiolipin (CL) molecular species in three healthy controls (CTRs) and Subject S1 (S1) skeletal muscle determined by mass spectrometry-based lipidome analysis. (**B**) Total cardiolipin levels measured in dry blood spots from 11 CTRs, Subjects S1 and S5. (**C**) Total cardiolipin abundance measured in primary fibroblasts from four CTRs, Subjects S1, S2 and S5. Error bars represent standard deviation; *n* = 3–8, where each data-point represents an independent biological sample.

To evaluate the effects of the *PTPMT1* variants on cardiolipin, we performed mass spectrometry-based cardiolipin species analysis as previously described.^45,46^ A reduction of approximately 70% in the 72:8 cardiolipin molecular species compared to healthy controls was observed in the skeletal muscle tissue of Subject S1 (Fig. 4A). Analysis of the dried blood spots of Subjects S1 and S5 revealed a reduction in cardiolipin content (Fig. 4B). Cardiolipin modulates complex I activity and substrate accessibility to the active site of the enzyme.^47,48^ Consistent with these observations, Subject S1 had decreased Complex I activity in skeletal muscle homogenate (Supplementary Table 5). Notably, we did not observe any reduction in total cardiolipin content in Subject S1 cultured fibroblasts. No skeletal muscle was available for Subjects S2 and S5. However, total cardiolipin levels were decreased in the cultured fibroblasts of Subject S5 (Fig. 4C). These observations correlated with the severity of the clinical phenotype; Subject S5 had a more severe neurological disorder compared to Subject S1 (Table 1). The milder clinical phenotype reported for Subject S1 could explain why the cultured fibroblasts of Subject S1 failed to display an aberrant cardiolipin profile. Overall, these data confirmed impaired cardiolipin biosynthesis in the three probands and reflected the biochemical and clinical severity of the disease. These results support the pathogenic effects of the *PTPMT1* variants and confirm that protein loss is associated with a significant reduction in total cardiolipin levels.

### *PTPMT1* variants induce loss of protein and mitochondrial fragmentation

**Figure 5:**
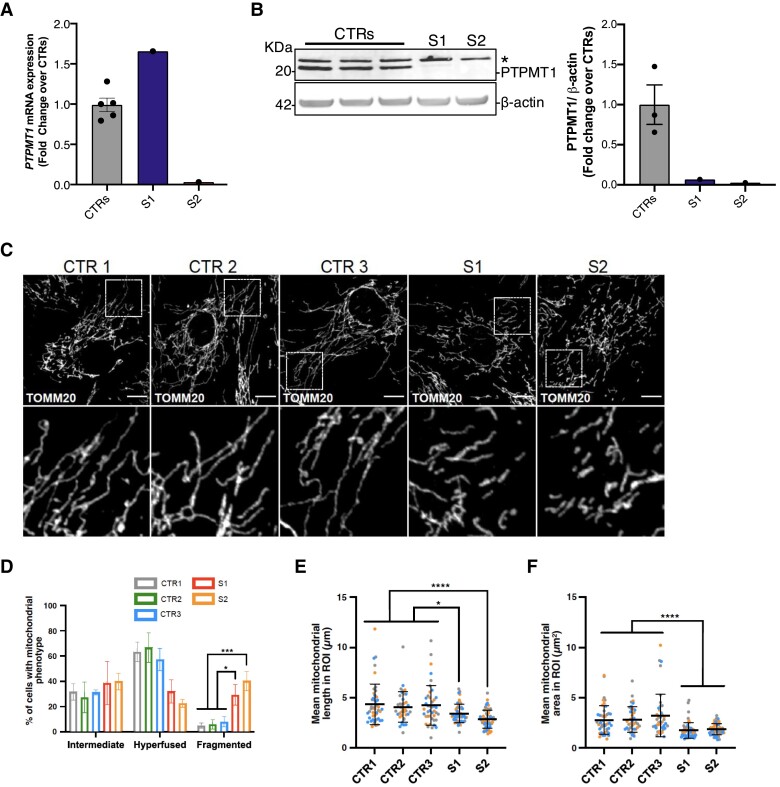
**Mitochondrial morphology in patient-derived fibroblasts.** (**A**) Relative expression of *PTPMT1* mRNA in primary fibroblasts analysed by quantitative PCR. No decrease was observed in Subject S1 (S1), whereas Subject S2 (S2) showed a severe loss of *PTPMT1* mRNA. Error bars represent standard error of the mean; *n* = 5, where each data-point represents an independent biological sample. (**B**) Western blot detecting the PTPMT1 protein expression in primary fibroblasts from three healthy controls (CTRs), Subjects S1 and S2. An asterisk indicates a non-specific signal. β-Actin was used as a loading control. (**C**) Representative confocal images of mitochondrial morphology from three healthy CTRs, Subjects S1 and S2. Mitochondria were labelled with an anti-TOMM20 antibody. Scale bar = 10 μm. (**D**) Quantification of mitochondrial morphology related to **C**. (**E** and **F**) Quantification of mitochondrial length (**E**) and area (**F**), per region of interest (ROI) (225 µm^2^) from **D**. Data are expressed as mean ± standard error of the mean (SEM) (**A**) or standard deviation (**D–F**); *n* = 3 independent experiments. **D**: Two-way ANOVA, Tukey’s multiple comparison test; **E** and **F**: unpaired Mann–Whitney U-test (two-tailed). *P < 0.05, **P < 0.01, ***P < 0.001.

To determine if both *PTPMT1* variants were associated with decreased *PTPMT1* transcript abundance, we measured mRNA relative abundance by qPCR using cDNA from the cultured fibroblasts of Subjects S1, S2 and S5. Transcript abundance was unchanged in Subject S1 (Fig. 5A). A severe reduction in *PTPMT1* mRNA levels was detected in Subjects S2 and S5, compared to five controls (Fig. 5A and Supplementary Fig. 4A), which was corroborated by RNA-seq data (Supplementary Fig. 3C). To investigate the effects of the *PTPMT1* variants on protein expression and stability, we performed western blot analysis on total protein extracted from cultured fibroblasts. Western blot analysis revealed decreased steady-state levels of PTPMT1 in all three probands, compared to three healthy controls (Fig. 5B and Supplementary Fig. 4B). This suggested the *PTPMT1* variants affect protein stability in S1, while the reduction observed in Subjects S2 and S5 is caused by the aberrant splicing event and NMD.

A quantitative loss of OXPHOS subunit expression, including NDUFB8 (Complex I), SDHB (Complex II) and COXII (Complex IV), was observed in the cultured fibroblasts of Subjects S1 and S2 during early passages (Supplementary Fig. 4C); however, this phenotype was not retained into later passage numbers, consistent with the reported limitations of using fibroblasts to investigate mitochondrial function.^49^ The detrimental effects of aberrant PTPMT1 expression on OXPHOS activity were confirmed by respiratory chain enzyme analysis performed on the skeletal muscle tissue of Subject S1 (Supplementary Table 5). No changes in mtDNA content were observed in fibroblasts from Subjects S1 and S2 (Supplementary Fig. 4D and E). Overall, our data suggested that decreased or absent levels of PTPMT1 protein impact cardiolipin content and impair OXPHOS activity, which likely contributes to the clinical phenotype observed in affected subjects.

**Figure 6:**
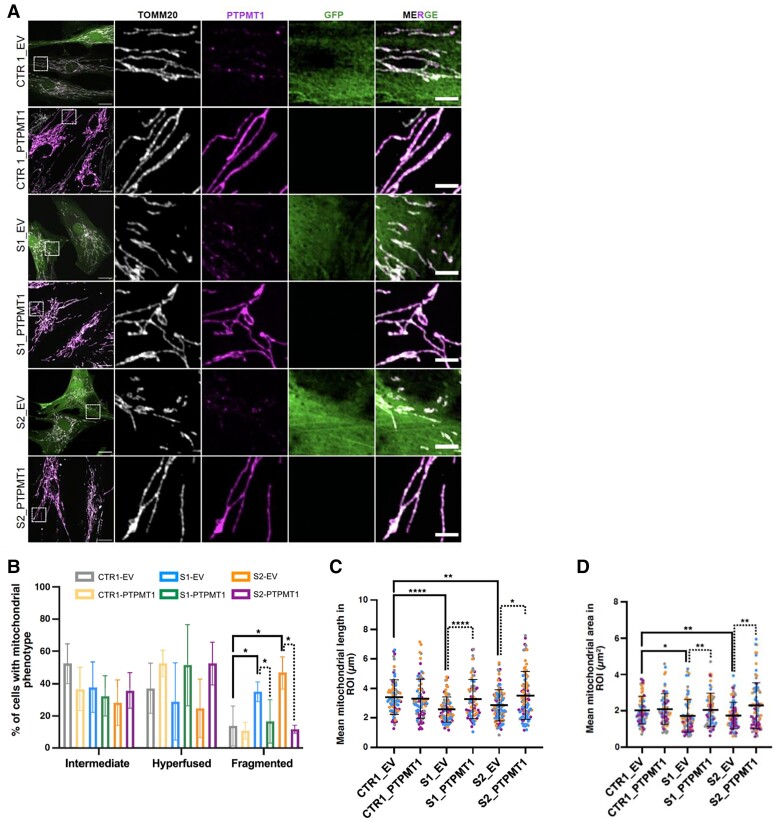
**PTPMT1 expression and mitochondrial morphology in complemented patient-derived fibroblasts.** (**A**) Representative confocal images of mitochondrial morphology and PTPMT1 expression in a healthy control (CTR1), Subject S1 (S1) and Subject S2 (S2) before and after transduction with pLenti6.3/V5-DEST-GFP (empty vector, EV) or pLenti6.3/V5-DEST vector expressing wild-type *PTPMT1*. Mitochondria and PTPMT1 were labelled with anti-TOMM20 and anti-PTPMT1 antibodies, respectively. Scale bar = 10 μm. (**B**) Quantification of mitochondrial morphology related to **A**. (**C** and **D**) Quantification of mitochondrial length (**C**) and area (**D**) per region of interest (ROI) (225 µm^2^) from **B**. Data are expressed as mean ± standard deviation; *n* = 4 independent experiments. **B**: Two-way ANOVA, Tukey’s multiple comparison test; **C** and **D**: unpaired Mann–Whitney U-test (two-tailed). *P < 0.05, **P < 0.01, ***P < 0.001.

Cardiolipin is involved in maintaining membrane architecture and mitochondrial morphology.^50^ We, therefore, investigated the effects of the PTPMT1 variants on mitochondrial morphology using confocal microscopy analysis of mutant PTPMT1 patient-derived fibroblasts. Subjects S1 and S2 fibroblasts harboured fragmented mitochondria, characterized by an increased number of cells with small round-shaped mitochondria and a decrease of mitochondrial length and area compared to controls (Fig. 5C–F). Importantly, aberrant mitochondrial morphology was rescued using stable expression of wild-type PTPMT1 in the cultured fibroblasts of Subjects S1 and S2 (Fig. 6A and B). In particular, the number of cells harbouring small round-shaped mitochondria and the decrease in organelle length and area observed in patient fibroblasts was significantly rescued in the complemented cells to levels similar to control lines (Fig. 6C and D). These data indicated that loss of PTPMT1 alters the mitochondrial fission-fusion balance, thereby inducing mitochondrial fragmentation. This phenotype was rescued by complementing patient-derived cells with wild-type *PTPMT1*, thus confirming the pathogenicity of the variants.

### Phenotype of *ptpmt1* knockout zebrafish

**Figure 7:**
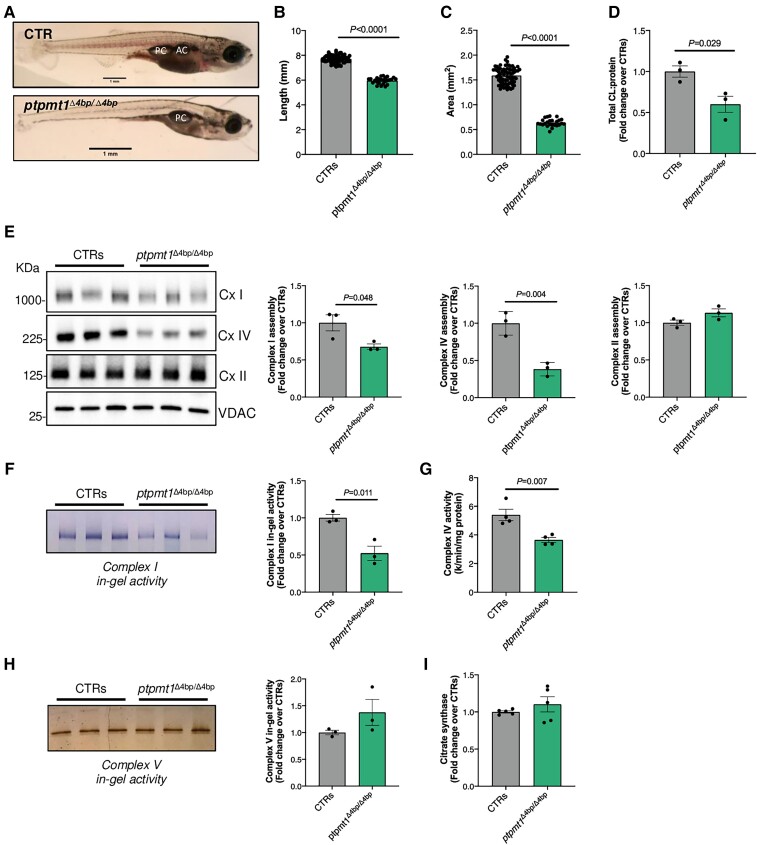
**Morphological, cardiolipin content, and oxidative phosphorylation complex analysis of *ptpmt1* knockout zebrafish.** (**A**) Representative image of *ptpmt1*^+/+^ (CTR) and *ptpmt*^Δ4bp/Δ4bp^. (**B**) Quantification of zebrafish body length. (**C**) Quantification of zebrafish head area. (**D**) Total cardiolipin (CL) levels measured by mass spectrometry-based lipidome analysis. (**E**) Western blot analysis of blue-native polyacrylamide gels loaded with 23.8 μg of protein extracted from a pool of two CTRs and 10 *ptpmt*^Δ4bp/Δ4bp^ zebrafish samples. (**F**) In-gel activity of Complex (Cx) I in a polyacrylamide blue-native gel loaded with 35.7 μg of protein extracted from a pool of two CTRs and 10 *ptpmt*^Δ4bp/Δ4bp^ zebrafish samples. (**G**) Spectrophotometric enzyme activity of Complex IV in CTRs and 10 *ptpmt*^Δ4bp/Δ4bp^ zebrafish samples. (**H**) In-gel activity of Complex V in a polyacrylamide clear-native gel loaded with 11.4 μg of protein extracted from a pool of two CTRs and 10 *ptpmt*^Δ4bp/Δ4bp^ zebrafish samples. (**I**) Citrate synthase activity measured in CTRs and *ptpmt*^Δ4bp/Δ4bp^. All measures were performed at 19 days post fertilization (dpf). The mutant allele is referred to as *ptpmt1*^Δ4bp^. Control groups (CTRs) were obtained by pooling together *ptpmt1*^+/+^ and *ptpmt*^+/Δ4bp^ samples. Error bars represent standard error of the mean, *n* = 3–71, where each data-point represents an independent biological sample. Significance values are shown for *t*-test compared to CTRs.

To evaluate the effect of loss of PTPMT1 *in vivo*, we generated a stable *ptpmt1* knockout zebrafish model using CRISPR-Cas9 technology. The mutation target site was chosen in exon 3, as this was the highest-scoring site according to CHOPCHOP (https://chopchop.cbu.uib.no). Exon 1 was avoided to prevent an alternative translation start site from being chosen or for the affected exon potentially to be spliced out. In the F1 generation, a 4 bp deletion was identified in exon 3, which was predicted to introduce a premature stop codon, 16 amino acids downstream of the mutation site (Supplementary Fig. 6A). The mutant allele is referred to as *ptpmt1*^Δ4bp^. This mutation introduces a restriction site, which allows for digestion with the restriction endonuclease *Sex*AI for genotyping. It was not possible to quantify the abundance of Ptpmt1 protein due to the lack of a suitable antibody in the zebrafish model. mRNA analysis indicated that there is a significant reduction in the level of *ptpmt1* mRNA in 19 dpf *ptpmt1*^Δ4bp/4bp^ fish relative to the control group obtained by pooling together *ptpmt1*^+/+^ and *ptpmt1*^+/Δ4bp^ 19 dpf fishes (*P* = 0.009) (Supplementary Fig. 6B). There was no morphological difference between *ptpmt1*^+/+^, *ptpmt1*^+/Δ4bp^ and *ptpmt1*^Δ4bp/4bp^ larvae <5 dpf. However, loss of *ptpmt1* caused a significant reduction of body and head size in a 19 dpf *ptpmt1*^Δ4bp/4bp^ zebrafish compared to their *ptpmt1*^+/+^ and *ptpmt1*^+/Δ4bp^ clutch mates (Fig. 7A–C). In addition, all 19 dpf *ptpmt1*^Δ4bp/4bp^ fish failed to inflate the anterior chamber (AC) of their swim bladders (Fig. 7A), suggesting that Ptpmt1 protein plays an essential role in development, as previously reported for other *in vivo* models.^19,24,51^ Notably, cardiolipin analysis by mass-spectrometry revealed that cardiolipin (72:8) levels, the most abundant cardiolipin species, were significantly diminished in the *ptpmt1*^Δ4bp/4bp^ zebrafish at 19 dpf (Fig. 7D), consistent with the key role of the protein in cardiolipin *de novo* biosynthesis. To investigate the effects of impaired cardiolipin content on the mitochondrial respiratory chain complex in *ptpmt1* knockout zebrafish, blue-native gel electrophoresis was undertaken on protein extracted from 19 dpf *ptpmt1*^Δ4bp/4bp^ fish, which confirmed a reduction in fully assembled Complex I (NADH dehydrogenase) and Complex IV (cytochrome c oxidase) in the *ptpmt1*^Δ4bp/4bp^ fish, compared with controls (Fig. 7E and Supplementary Fig. 7A–C). There was increased anti-NDUFS3 immunoreactivity at 30 kDa in the *ptpmt1*^Δ4bp/4bp^ fish, suggesting accumulation of free, unassembled NDUFS3 (Supplementary Fig. 7A). Complex II assembly was normal (Fig. 7E). In-gel activity staining of blue-native gel electrophoresis gels revealed a 50% decrease in Complex I activity (Fig. 7F). Spectrophotometric activity confirmed that Complex IV activity was reduced by 30% in the *ptpmt1*^Δ4bp/4bp^ fish (Fig. 7G). These data are consistent with the known role of cardiolipin in Complex I and IV stability and activity.^48,52^ In-gel activity of Complex V was normal (Fig. 7H). Additionally, there were no differences in the activity of citrate synthase (Fig. 7I), widely used as a biomarker of mitochondrial content.^53,54^ Taken together, these data suggest that loss of *ptpmt1* decreases total cardiolipin content, disrupting mitochondrial respiratory chain assembly and activity, resulting in developmental and cellular maturation abnormalities in zebrafish at 19 dpf.

## Discussion

Cardiolipin has recently become an important area of neurological disease research. Aberrant cardiolipin content, structure and localization are linked with impaired neurogenesis and neuronal dysfunction, contributing to ageing and the pathophysiology of several neurodegenerative disorders, including idiopathic Parkinson’s disease, Alzheimer’s disease and amyotrophic lateral sclerosis.^5^ One emerging category of PMDs, which are associated with cognitive and neurological manifestations, is caused by pathogenic variants in cardiolipin biosynthesis and maintenance genes.

In this study, we describe six individuals (Subjects S1–S6) from three unrelated families harbouring biallelic variants in *PTPMT1* with a neonatal/infantile onset with variable severity. Subject S1 displayed mild intellectual disability associated with thinning of the corpus callosum and atrophy of the pons and medulla, while Subjects S2–S5 were more severely affected with microcephaly, ataxia, increased tone and white matter changes on brain MRI. Importantly, Subject S6 shared the same *PTPMT1* variant as Subjects S2–S5 but was less severely affected. PMDs are typified by variability in their clinical presentation, which may be influenced by environmental and background genetic effects. Potentially, Subject S6 might benefit from protective factors that confer a milder phenotype. Alternatively, the clinical syndrome could further progress with age and become comparable in severity to Subjects S2–S5, given that he is younger than these subjects. Notably, although brain MRI was normal at 9 months, he subsequently developed symptoms at 1 year.

Using next-generation sequencing, we identified two homozygous variants, one missense (Family 1) and one missense/splice region variant (Families 2 and 3), which segregated with affected individuals. We hypothesize that both reported *PTPMT1* variants lead to loss of function, although minimal PTPMT1 expression was retained in Subject S1 and correlated with a milder disease phenotype. Further evidence for a loss-of-function hypothesis includes: (i) decreased total cardiolipin content in skeletal muscle and dry blood spot; (ii) abnormal mitochondrial morphology, with increased fragmentation of mitochondria in fibroblasts; (iii) a cellular phenotype in fibroblasts that was complemented by exogenous expression of wild-type PTPMT1; and (iv) a *ptpmt1* knockout zebrafish model associated with abnormalities in body size, developmental alterations, decreased total cardiolipin levels and altered OXPHOS complex assembly and activity, findings consistent with previous studies highlighting the important contribution of cardiolipin during cellular proliferation and postnatal development.^24-26^

Consistent with the central role of PTPMT1 in cardiolipin biosynthesis, reduced cardiolipin (72:8) levels were detected in the muscle tissue and blood from Subject S1 and were associated with low Complex I activity in muscle. Reduced levels of total cardiolipin were detected in the fibroblasts of Subjects S2 and S5 (missense/splice region variant), while the fibroblasts of Subject S1 (missense variant) exhibited normal total cardiolipin content (Fig. 4C and Supplementary Fig. 4F). Several *in vitro* and *in vivo* studies suggest that cardiolipin content and acyl chain composition are tissue-specific.^55,56^ Similar findings are reported in other cardiolipin-related disorders, including *TAMM41* and *CRLS1* patient-derived fibroblasts, which failed to demonstrate abnormal cardiolipin profiles. We propose the tissue-specific cardiolipin effects of the two *PTPMT1* variants reflect a hypomorphic allele in Subject S1 (missense variant) and a more deleterious loss-of-function variant in Subjects S2 and S5. This reasoning is underpinned by RNA analysis, which revealed that the c.255G>C variant was associated with aberrant splicing and NMD, resulting in loss of PTPMT1 protein expression, contrary to the less severe PTPMT1 protein reduction associated with the missense variant. The small number of subjects precludes conclusive genotype-phenotype correlations, although subjects with the splice site variant had more severe neurological manifestations, including microcephaly and ataxia. Consequently, we suggest that dry blood spots and skeletal muscle tissue are more sensitive to detecting reduced total cardiolipin content in individuals with milder clinical presentations. Analysis of fibroblasts revealed that loss of PTPMT1 was associated with fragmented mitochondria. Notably, abnormal mitochondrial morphology was observed in the Subject S1 (c.65A>C) and Subject S2 (c.255G>C) patient cell lines; this is a common finding of *in vitro* and *in vivo PTPMT1* knockout models.^19,24^ The altered morphological features observed in the *PTPMT1* patient fibroblasts are consistent with the regulatory role of cardiolipin in mitochondria morphology and dynamics.^57^ Similarly, other cardiolipin monogenic disorders (e.g. patient-derived mutant *CRLS1* fibroblasts and *TAZ* lymphoblasts) exhibit increased mitochondrial fragmentation.^16,58^ The morphological defects caused by the *PTPMT1* variants were rescued by overexpressing wild-type *PTPMT1* in the patient-derived fibroblasts, thus confirming the pathogenic effects of the two variants.

Cardiolipin is a key component of the IMM that facilitates the assembly and stability of the OXPHOS protein complexes.^47,52,59,60^ To understand the effect of PTPMT1 deficiency and consequent decreased cardiolipin on the respiratory complexes, we performed blue-native gel electrophoresis and mitochondrial respiratory chain enzyme activity measurements in a zebrafish model. Ablation of *ptpmt1* had a profound effect on OXPHOS function, resulting in reduced Complex I and IV assembly and activity. These results are consistent with the findings from the skeletal muscle tissue of Subject S1, which revealed a significant decrease in Complex I activity. Complex IV activity was also at the lower end of the reference range despite Complex II and III activities being at the high end. The similarity in OXPHOS defects observed in the skeletal muscle of Subject S1 and the zebrafish model indicates that PTPMT1 is essential for the optimal stability, assembly and enzymatic activity of these complexes. Given the importance of cardiolipin in the structural organization and stabilization of the mitochondrial respiratory chain supercomplexes,^61,62^ further work is required to investigate the consequences of mutant PTPMT1 on supercomplex organization within the electron transport chain.

Major limitations of the current study include the lack of patient-derived skeletal muscle tissue to investigate the effects of the *PTPMT1* variants.^49^ Cardiolipin measurements in fibroblasts, a particularly accessible patient-derived tissue, failed to consistently demonstrate alterations in total cardiolipin and acyl chain composition or altered expression of OXPHOS subunits. A mutant *in vivo* model was therefore required to further elucidate the role of PTPMT1 deficiency in total cardiolipin content and OXPHOS function. In future, it would be valuable to evaluate the role of mutant PTPMT1 in neuronal cells reprogrammed from patient-derived fibroblasts using induced pluripotent stem cell (iPSC) technology.^63^ Such iPSC-based modelling would help further characterize the role of PTPMT1 in maintaining mitochondrial function within a relevant target tissue. Finally, six individuals from three unrelated families were identified. Although sufficient to establish *PTPMT1* as a new disease gene and undertake an extensive molecular characterization, the small sample size does not enable a complete understanding of the phenotypic spectrum of PTPMT1-related diseases. Consequently, identification and collation of additional pathogenic *PTPMT1* variants will be crucial to comprehensively characterize the disease spectrum.

In summary, we established *PTPMT1* as a new cardiolipin-related PMD gene and provide evidence that supports a role for cardiolipin during neurodevelopment. This discovery also contributes to the clinical and genetic spectrum of Mendelian disorders associated with aberrant cardiolipin biosynthesis and acyl chain remodelling. Such phenotypes range from severe myopathy, cardiomyopathy and neutropenia (*TAZ*) to multisystemic disorders comprising progressive encephalopathy and neurodevelopmental regression (*PNPLA8*, *CRLS1*, *TAMM41*, *PTPMT1*).^10,16,17,64^ Cardiolipin is particularly abundant in heart tissue; however, cardiac manifestations were not observed in the herein reported subjects. This finding is consistent with previous reports of other cardiolipin-related disease genes (*TAMM41*).^17^ Importantly, several aspects of cardiolipin metabolism are not fully understood, including the factors that regulate the tissue-specific acyl chain composition of cardiolipin and the contribution of cardiolipin-related genes to human pathophysiology. Consequently, as further cardiolipin-related genes emerge, research should attempt to better understand the variable clinical manifestations linked to abnormal cardiolipin species.

## Supplementary Material

awae268_Supplementary_Data

## Data Availability

The authors confirm that the data supporting the findings of this study are available within the article and/or its Supplementary material. These data are available from the corresponding author upon request.

## References

[1] Gorman GS, Schaefer AM, Ng Y, et al Prevalence of nuclear and mitochondrial DNA mutations related to adult mitochondrial disease. Ann Neurol. 2015;77:753–759. 25652200 10.1002/ana.24362 PMC4737121

[2] Chinnery PF. Primary mitochondrial disorders overview. In: Adam MP, Feldman J, Mirzaa GM, et al., eds. *GeneReviews®*. University of Washington; 1993-2024. https://www.ncbi.nlm.nih.gov/books/NBK1224/20301403

[3] Lu YW, Claypool SM. Disorders of phospholipid metabolism: An emerging class of mitochondrial disease due to defects in nuclear genes. Front Genet. 2015;6:3. 25691889 10.3389/fgene.2015.00003 PMC4315098

[4] Keller MA . Interpreting phospholipid and cardiolipin profiles in rare mitochondrial diseases. Curr Opin Syst Biol. 2021;28:100383.

[5] Falabella M, Vernon HJ, Hanna MG, Claypool SM, Pitceathly RDS. Cardiolipin, mitochondria, and neurological disease. Trends Endocrinol Metab. 2021;32:224–237. 33640250 10.1016/j.tem.2021.01.006 PMC8277580

[6] Kameoka S, Adachi Y, Okamoto K, Iijima M, Sesaki H. Phosphatidic acid and cardiolipin coordinate mitochondrial dynamics. Trends Cell Biol. 2018;28:67–76. 28911913 10.1016/j.tcb.2017.08.011 PMC5742555

[7] Quintana-Cabrera R, Scorrano L. Determinants and outcomes of mitochondrial dynamics. Mol Cell. 2023;83:857–876. 36889315 10.1016/j.molcel.2023.02.012

[8] Tilokani L, Nagashima S, Paupe V, Prudent J. Mitochondrial dynamics: Overview of molecular mechanisms. Essays Biochem. 2018;62:341–360. 30030364 10.1042/EBC20170104 PMC6056715

[9] Bione S, D’Adamo P, Maestrini E, Gedeon AK, Bolhuis PA, Toniolo D. A novel X-linked gene, G4.5. is responsible for Barth syndrome. Nat Genet. 1996;12: 385–389. 8630491 10.1038/ng0496-385

[10] Barth PG, Scholte HR, Berden JA, et al An X-linked mitochondrial disease affecting cardiac muscle, skeletal muscle and neutrophil leucocytes. J Neurol Sci. 1983;62: 327–355. 6142097 10.1016/0022-510x(83)90209-5

[11] Mayr JA, Haack TB, Graf E, et al Lack of the mitochondrial protein acylglycerol kinase causes sengers syndrome. Am J Hum Genet. 2012;90: 314–320. 22284826 10.1016/j.ajhg.2011.12.005 PMC3276657

[12] Saunders CJ, Moon SH, Liu X, et al Loss of function variants in human PNPLA8 encoding calcium-independent phospholipase A2 γ recapitulate the mitochondriopathy of the homologous null mouse. Hum Mutat. 2015;36:301–306. 25512002 10.1002/humu.22743 PMC4361307

[13] Nakamura Y, Shimada IS, Maroofian R, et al Biallelic null variants in PNPLA8 cause microcephaly by reducing the number of basal radial glia. Brain. 2024;147:3949–3967. 39082157 10.1093/brain/awae185 PMC11531855

[14] Davey KM, Parboosingh JS, McLeod DR, et al Mutation of DNAJC19, a human homologue of yeast inner mitochondrial membrane co-chaperones, causes DCMA syndrome, a novel autosomal recessive Barth syndrome-like condition. J Med Genet. 2006;43: 385–393. 16055927 10.1136/jmg.2005.036657 PMC2564511

[15] Wortmann SB, Vaz FM, Gardeitchik T, et al Mutations in the phospholipid remodeling gene SERAC1 impair mitochondrial function and intracellular cholesterol trafficking and cause dystonia and deafness. Nat Genet. 2012;44: 797–802. 22683713 10.1038/ng.2325

[16] Lee RG, Balasubramaniam S, Stentenbach M, et al Deleterious variants in CRLS1 lead to cardiolipin deficiency and cause an autosomal recessive multi-system mitochondrial disease. Hum Mol Genet. 2022;31:3597–3612. 35147173 10.1093/hmg/ddac040 PMC9616573

[17] Thompson K, Bianchi L, Rastelli F, et al Biallelic variants in TAMM41 are associated with low muscle cardiolipin levels, leading to neonatal mitochondrial disease. HGG Adv. 2022;3:100097. 35321494 10.1016/j.xhgg.2022.100097 PMC8935507

[18] Pagliarini DJ, Worby CA, Dixon JE. A PTEN-like phosphatase with a novel substrate specificity. J Biol Chem. 2004;279:38590–38596. 15247229 10.1074/jbc.M404959200

[19] Zhang J, Guan Z, Murphy AN, et al Mitochondrial phosphatase PTPMT1 is essential for cardiolipin biosynthesis. Cell Metab. 2011;13:690–700. 21641550 10.1016/j.cmet.2011.04.007 PMC3119201

[20] Xiao J, Engel JL, Zhang J, Chen MJ, Manning G, Dixon JE. Structural and functional analysis of PTPMT1, a phosphatase required for cardiolipin synthesis. Proc Natl Acad Sci U S A. 2011;108:11860–11865. 21730175 10.1073/pnas.1109290108 PMC3142007

[21] Pagliarini DJ, Wiley SE, Kimple ME, et al Involvement of a mitochondrial phosphatase in the regulation of ATP production and insulin secretion in pancreatic β cells. Mol Cell. 2005;19:197–207. 16039589 10.1016/j.molcel.2005.06.008

[22] Shen J, Liu X, Yu W-M, et al A critical role of mitochondrial phosphatase Ptpmt1 in embryogenesis reveals a mitochondrial metabolic stress-induced differentiation checkpoint in embryonic stem cells. Mol Cell Biol. 2011;31:4902–4916. 21986498 10.1128/MCB.05629-11 PMC3233018

[23] Zheng H, Li Q, Li S, et al Loss of Ptpmt1 limits mitochondrial utilization of carbohydrates and leads to muscle atrophy and heart failure in tissue-specific knockout mice. Elife. 2023;12:e86944. 10.7554/eLife.86944 PMC10482430 37672386

[24] Zhu S, Wang H, Wang L, et al PTPMT1 is required for embryonic cardiac cardiolipin biosynthesis to regulate mitochondrial morphogenesis and heart development. Circulation. 2021;144:403–406. 34339306 10.1161/CIRCULATIONAHA.121.054768 PMC8340985

[25] Olivar-Villanueva M, Ren M, Schlame M, Phoon CKL. The critical role of cardiolipin in metazoan differentiation, development, and maturation. Dev Dyn. 2023;252: 691–712. 36692477 10.1002/dvdy.567 PMC10238668

[26] Zheng H, Yu WM, Shen J, et al Mitochondrial oxidation of the carbohydrate fuel is required for neural precursor/stem cell function and postnatal cerebellar development. Sci Adv. 2018;4:eaat2681. 30338292 10.1126/sciadv.aat2681 PMC6191298

[27] Turnbull C, Scott RH, Thomas E, et al The 100 000 genomes project: Bringing whole genome sequencing to the NHS. BMJ. 2018;361:k1687. 29691228 10.1136/bmj.k1687

[28] Hiz Kurul S, Oktay Y, Töpf A, et al High diagnostic rate of trio exome sequencing in consanguineous families with neurogenetic diseases. Brain. 2022;145:1507–1518. 34791078 10.1093/brain/awab395 PMC9128813

[29] Richards S, Aziz N, Bale S, et al Standards and guidelines for the interpretation of sequence variants: A joint consensus recommendation of the American College of Medical Genetics and genomics and the association for molecular pathology. Genet Med. 2015;17:405–424. 25741868 10.1038/gim.2015.30 PMC4544753

[30] Dobin A, Davis CA, Schlesinger F, et al STAR: Ultrafast universal RNA-seq aligner. Bioinformatics. 2013;29:15–21. 23104886 10.1093/bioinformatics/bts635 PMC3530905

[31] Ewels P, Magnusson M, Lundin S, Käller M. MultiQC: Summarize analysis results for multiple tools and samples in a single report. Bioinformatics. 2016;32:3047–3048. 27312411 10.1093/bioinformatics/btw354 PMC5039924

[32] Pimentel H, Bray NL, Puente S, Melsted P, Pachter L. Differential analysis of RNA-seq incorporating quantification uncertainty. Nat Methods. 2017;14:687–690. 28581496 10.1038/nmeth.4324

[33] Varshney GK, Carrington B, Pei W, et al A high-throughput functional genomics workflow based on CRISPR/Cas9-mediated targeted mutagenesis in zebrafish. Nat Protoc. 2016;11:2357–2375. 27809318 10.1038/nprot.2016.141 PMC5630457

[34] Labun K, Montague TG, Krause M, Torres Cleuren YN, Tjeldnes H, Valen E. CHOPCHOP v3: Expanding the CRISPR web toolbox beyond genome editing. Nucleic Acids Res. 2019;47:W171–W174. 31106371 10.1093/nar/gkz365 PMC6602426

[35] Schindelin J, Arganda-Carreras I, Frise E, et al Fiji: An open-source platform for biological-image analysis. Nat Methods. 2012;9:676–682. 22743772 10.1038/nmeth.2019 PMC3855844

[36] Schägger H . Quantification of oxidative phosphorylation enzymes after blue native electrophoresis and two-dimensional resolution: Normal complex I protein amounts in Parkinson’s disease conflict with reduced catalytic activities. Electrophoresis. 1995;16:763–770. 7588559 10.1002/elps.11501601125

[37] Capaldi RA, Marusich MF, Taanman JW. Mammalian cytochrome-c oxidase: Characterization of enzyme and immunological detection of subunits in tissue extracts and whole cells. Methods Enzymol. 1995;260:117–132. 8592440 10.1016/0076-6879(95)60134-1

[38] Zerbetto E, Vergani L, Dabbeni-Sala F. Quantification of muscle mitochondrial oxidative phosphorylation enzymes via histochemical staining of blue native polyacrylamide gels. Electrophoresis. 1997;18:2059–2064. 9420170 10.1002/elps.1150181131

[39] Wittig I, Karas M, Schägger H. High resolution clear native electrophoresis for in-gel functional assays and fluorescence studies of membrane protein complexes. Mol Cell Proteomics. 2007;6:1215–1225. 17426019 10.1074/mcp.M700076-MCP200

[40] Suhai T, Heidrich NG, Dencher NA, Seelert H. Highly sensitive detection of ATPase activity in native gels. Electrophoresis. 2009;30:3622–3625. 19784950 10.1002/elps.200900114

[41] Jumper J, Evans R, Pritzel A, et al Highly accurate protein structure prediction with AlphaFold. Nature. 2021;596:583–589. 34265844 10.1038/s41586-021-03819-2 PMC8371605

[42] Jaganathan K, Kyriazopoulou Panagiotopoulou S, McRae JF, et al Predicting splicing from primary sequence with deep learning. Cell. 2019;176:535–548.e24. 30661751 10.1016/j.cell.2018.12.015

[43] Yeo G, Burge CB. Maximum entropy modeling of short sequence motifs with applications to RNA splicing signals. J Comput Biol. 2004;11: 377–394. 15285897 10.1089/1066527041410418

[44] Ioannidis NM, Rothstein JH, Pejaver V, et al REVEL: An ensemble method for predicting the pathogenicity of rare missense variants. Am J Hum Genet. 2016;99:877–885. 27666373 10.1016/j.ajhg.2016.08.016 PMC5065685

[45] Vaz FM, van Lenthe H, Vervaart MAT, et al An improved functional assay in blood spot to diagnose Barth syndrome using the monolysocardiolipin/cardiolipin ratio. J Inherit Metab Dis. 2022;45:29–37. 34382226 10.1002/jimd.12425 PMC9291596

[46] Vaz FM, McDermott JH, Alders M, et al Mutations in PCYT2 disrupt etherlipid biosynthesis and cause a complex hereditary spastic paraplegia. Brain. 2019;142:3382–3397. 31637422 10.1093/brain/awz291 PMC6821184

[47] Jussupow A, Di Luca A, Kaila VRI. How cardiolipin modulates the dynamics of respiratory complex I. Sci Adv. 2019;5:eaav1850. 30906865 10.1126/sciadv.aav1850 PMC6426460

[48] Fiedorczuk K, Letts JA, Degliesposti G, Kaszuba K, Skehel M, Sazanov LA. Atomic structure of the entire mammalian mitochondrial complex I. Nature. 2016;538:406–410. 27595392 10.1038/nature19794 PMC5164932

[49] Acin-Perez R, Benincá C, Shabane B, Shirihai OS, Stiles L. Utilization of human samples for assessment of mitochondrial bioenergetics: Gold standards, limitations, and future perspectives. Life. 2021;11:949. 34575097 10.3390/life11090949 PMC8467772

[50] Schlame M, Ren M. The role of cardiolipin in the structural organization of mitochondrial membranes. Biochim Biophys Acta. 2009;1788:2080–2083. 19413994 10.1016/j.bbamem.2009.04.019 PMC2757492

[51] Homma Y, Toga K, Daimon T, Shinoda T, Togawa T. A mitochondrial phosphatase PTPMT1 is essential for the early development of silkworm, Bombyx mori. Biochem Biophys Res Commun. 2020;530:713–718. 32773109 10.1016/j.bbrc.2020.07.124

[52] Qin L, Hiser C, Mulichak A, Garavito RM, Ferguson-Miller S. Identification of conserved lipid/detergent-binding sites in a high-resolution structure of the membrane protein cytochrome c oxidase. Proc Natl Acad Sci U S A. 2006;103:16117–16122. 17050688 10.1073/pnas.0606149103 PMC1616942

[53] Renner K, Amberger A, Konwalinka G, Kofler R, Gnaiger E. Changes of mitochondrial respiration, mitochondrial content and cell size after induction of apoptosis in leukemia cells. Biochim Biophys Acta—Mol Cell Res. 2003;1642:115–123. 10.1016/s0167-4889(03)00105-8 12972300

[54] Larsen S, Nielsen J, Hansen CN, et al Biomarkers of mitochondrial content in skeletal muscle of healthy young human subjects. J Physiol. 2012;590:3349–3360. 22586215 10.1113/jphysiol.2012.230185 PMC3459047

[55] Oemer G, Koch J, Wohlfarter Y, et al Phospholipid acyl chain diversity controls the tissue-specific assembly of mitochondrial cardiolipins. Cell Rep. 2020;30:4281–4291.e4. 32209484 10.1016/j.celrep.2020.02.115

[56] Schlame M, Greenberg ML. Biosynthesis, remodeling and turnover of mitochondrial cardiolipin. Biochim Biophys Acta Mol Cell Biol Lipids. 2017;1862:3–7. 27556952 10.1016/j.bbalip.2016.08.010 PMC5125896

[57] Dudek J . Role of cardiolipin in mitochondrial signaling pathways. Front Cell Dev Biol. 2017;5:297929. 10.3389/fcell.2017.00090 PMC5626828 29034233

[58] Acehan D, Xu Y, Stokes DL, Schlame M. Comparison of lymphoblast mitochondria from normal subjects and patients with Barth syndrome using electron microscopic tomography. Lab Invest. 2006;87:40–48. 17043667 10.1038/labinvest.3700480 PMC2215767

[59] Spikes TE, Montgomery MG, Walker JE. Structure of the dimeric ATP synthase from bovine mitochondria. Proc Natl Acad Sci U S A. 2020;117:23519–23526. 32900941 10.1073/pnas.2013998117 PMC7519299

[60] Palsdottir H, Lojero CG, Trumpower BL, Hunte C. Structure of the yeast cytochrome bc1 complex with a hydroxyquinone anion Qo site inhibitor bound. J Biol Chem. 2003;278:31303–31311. 12782631 10.1074/jbc.M302195200

[61] Pfeiffer K, Gohil V, Stuart RA, et al Cardiolipin stabilizes respiratory chain supercomplexes. J Biol Chem. 2003;278:52873–52880. 14561769 10.1074/jbc.M308366200

[62] Zhang M, Mileykovskaya E, Dowhan W. Gluing the respiratory chain together. Cardiolipin is required for supercomplex formation in the inner mitochondrial membrane. J Biol Chem. 2002;277:43553–43556. 12364341 10.1074/jbc.C200551200

[63] Tolle I, Tiranti V, Prigione A. Modeling mitochondrial DNA diseases: From base editing to pluripotent stem-cell-derived organoids. EMBO Rep. 2023;24:e55678. 36876467 10.15252/embr.202255678 PMC10074100

[64] Shukla A, Saneto RP, Hebbar M, Mirzaa G, Girisha KM. A neurodegenerative mitochondrial disease phenotype due to biallelic loss-of-function variants in PNPLA8 encoding calcium-independent phospholipase A2γ. Am J Med Genet A. 2018;176:1232–1237. 29681094 10.1002/ajmg.a.38687 PMC5918416

